# Pilot Observational Study to Detect Diurnal Variation and Misalignment in Heart Rate Among Critically Ill Patients

**DOI:** 10.3389/fneur.2020.00637

**Published:** 2020-07-15

**Authors:** Melissa P. Knauert, Terrence E. Murphy, Margaret M. Doyle, Margaret A. Pisani, Nancy S. Redeker, Henry K. Yaggi

**Affiliations:** ^1^Section of Pulmonary, Critical Care, and Sleep Medicine, Yale School of Medicine, New Haven, CT, United States; ^2^Section of Geriatrics, Yale School of Medicine, New Haven, CT, United States; ^3^Yale School of Nursing, West Haven, CT, United States

**Keywords:** sleep, circadian, diurnal, critical illness, misalignment, heart rate, intensive care unit

## Abstract

Circadian disruption is common in critically ill patients admitted to the intensive care unit (ICU). Understanding and treating circadian disruption in critical illness has significant potential to improve critical illness outcomes through improved cognitive, immune, cardiovascular, and metabolic function. Measurement of circadian alignment (i.e., circadian phase) can be resource-intensive as it requires frequent blood or urine sampling over 24 or more hours. Less cumbersome methods of assessing circadian alignment would advance investigations in this field. Thus, the objective of this study is to examine the feasibility of using continuous telemetry to assess diurnal variation in heart rate (HR) among medical ICU patients as a proxy for circadian alignment. In exploratory analyses, we tested for associations between misalignment of diurnal variation in HR and death during hospital admission. This was a prospective observational cohort study embedded within a prospective medical ICU biorepository. HR data were continuously collected (every 5 s) via telemetry systems for the duration of the medical ICU admission; the first 24 h of this data was analyzed. Patients were extensively characterized via medical record chart abstraction and patient interviews. Of the 56 patients with complete HR data, 48 (86%) had a detectable diurnal variation. Of these patients with diurnal variation, 39 (81%) were characterized as having the nadir of their HR outside of the normal range of 02:00–06:00 (“misalignment”). Interestingly, no deaths occurred in the patients with normally aligned diurnal variation; in contrast, there were seven deaths (out of 39 patients) in patients who had misaligned diurnal variation in HR. In an exploratory analysis, we found that the odds ratio of death for misaligned vs. aligned patients was increased at 4.38; however, this difference was not statistically significant (95% confidence interval 0.20–97.63). We conclude that diurnal variation in HR can be detected via continuous telemetric monitoring of critically ill patients. A majority of these patients with diurnal variation exhibited misalignment in their first 24 h of medical ICU admission. Exploratory analyses suggest possible associations between misalignment and death.

## Introduction

Circadian rhythms are one of nature's most pervasive adaptations allowing coordination between an organism and the earth's day-night environmental changes. Human circadian rhythms are self-sustaining oscillating processes driven by a central “master clock.” Adjustment of this master clock into alignment with day-night occurs via external cues. This process of adjusting the master clock is called *entrainment*, and the external cues capable of entrainment are termed *zeitgebers* (1). Key zeitgebers include light (2, 3), sleep (4, 5), meal timing (6, 7), social activity (8, 9), and exercise (10–12).

Circadian misalignment in the intensive care unit (ICU) is likely caused by multifactorial insults of acute critical illness with or without associated brain dysfunction, abnormal light exposure, continuous nutritional intake, immobility, and pleiotropic medication effects (13, 14). Circadian rhythm disruption overlaps and potentiates ICU sleep disruption forming a vicious cycle for critically ill patients. Sleep and circadian rhythm disruption are implicated in the development of ICU delirium (15–17). Days of delirium are associated with increased mortality, and sleep promotion protocols have been associated with decreases in delirium (18, 19). In addition, sleep and circadian disruption both are linked to impairments in immune, metabolic, and cardiovascular function (20–25). However, direct evidence in support of associations between circadian rhythm and outcomes relevant to critical illness outcomes is limited.

One limiting factor is difficulty with large scale measurement of circadian alignment (i.e., circadian phase). Melatonin is considered the gold standard as it directly reflects the phase of the master circadian clock. Measurement, via melatonin, of circadian alignment can be resource intensive because it requires frequent sampling over 24 or more hours. In addition, collection techniques require blood, urine, or saliva and are impacted by changes in light exposure, posture, food intake, or blood in the mouth (saliva samples) that can render samples inaccurate. Thus, in the ICU, the feasibility of measuring melatonin is significantly limited. Alternative assessments of the circadian rhythm may include leveraging markers of circadian rhythm, such as diurnal variations in heart rate (HR), blood pressure, and core body temperature. Humans have a stereotypic pattern of diurnal variation in HR with a normal nadir between 04:00 and 06:00 (26, 27), and HR is collected continuously for the vast majority of ICU patients.

The objective of this study is to examine the feasibility of using continuous telemetry to assess diurnal variation in HR among medical ICU patients as a proxy for circadian alignment. In exploratory analyses, we tested for associations between misalignment of diurnal variation in HR and select medical ICU outcomes.

## Methods

### Design, Study Population, and Setting

This was a prospective observational cohort study of critically ill patients. Patients were recruited from the medical ICU of a tertiary academic center. Patients were eligible for inclusion in a parent biorepository if they were 18 years or older and if the current medical ICU admission was the first medical ICU admission of the patient's hospital stay. Patients were excluded if they or their surrogate could not understand English sufficiently to participate in biorepository questionnaires, or if they were moribund, transferred from an outside hospital, or expected to leave the medical ICU in the 24 h following screening. Patients were eligible for inclusion in the HR cohort of the biorepository if they had been in the hospital <72 h prior to medical ICU admission and had complete HR data. Data capture of continuous HR became technically feasible in March 2016. Patients in our study were enrolled between March 2016 and June 2017.

### Ethics

Patient consent was obtained, and all study activities were approved by the Yale Institutional Review Board (IRB 1508016346).

### Measurements

HR data was extracted from the hospital telemetry systems, which provided beats per minute every 5 s. HR data analysis was limited to the first 24 h spent in the ICU. Patient-hours with fewer than 15 min of data were excluded from the analysis.

Patient data were collected via a review of the electronic medical record and patient/surrogate interview. The following data were collected: demographics, medical history, sleep history including the Pittsburgh Sleep Quality Index, home medications, the reason for ICU admission, whether systemic infection or sepsis was suspected, ICU treatments, and laboratory values. Patient data was used to calculate the acute physiology and chronic health evaluation II score (APACHE II) which estimates severity of illness in terms of predicted mortality using both acute and chronic health variables (28). Vasopressor use was tracked among patients to assess whether or not vasopressors which can alter HR would obscure the detection of diurnal HR variation.

### Feasibility of Detecting Diurnal Variation

Feasibility was assessed based on the percentage of patients having complete data and as the percentage of patients having a detectable pattern of diurnal variation based on cosinor analysis as described below.

### Heartrate Cosinor Analysis

Average hourly HRs for the first 24 h post-ICU admission were calculated and assessed for rhythmicity using a cosinor model (29). Briefly, the cosinor analysis fits a cosine curve to each patient's longitudinal data, which is hypothesized to demonstrate a cyclical pattern. Assuming a fixed time period, the cosine curve can be re-parameterized to facilitate the fitting of a linear regression model. Rejection of the null hypothesis of the overall model demonstrates that the cosine curve provides a better fit to the data than a mean only model and can be interpreted as evidence for diurnal variation in the longitudinal data. Subsequent statistical analyses were restricted to only those patients whose individual cosinor analyses provided evidence of diurnal variation.

The fitted cosinor model allows for the calculation of the mesor, amplitude, and nadir of the underlying cosine curve. The mesor is the average HR. The amplitude is the difference between the average and maximum HRs. The nadir is the time corresponding to minimal HR as estimated from each patient's individual cosinor curve.

### Analysis of Alignment of Heart Rate Nadir

Patients who had cosinor determined diurnal variation and a nadir falling between 02:00 and 06:00 were defined as being aligned. Those with a nadir outside this expected range were defined as being misaligned. Differences among groups (aligned, misaligned, no diurnal variation) in patient characteristics were tested with the Kruskal-Wallis, ANOVA or Fisher's Exact tests as appropriate. In addition, in exploratory analyses, unadjusted logistic regression was used to evaluate the strength of association between selected explanatory variables, including misalignment with death during the patient's stay in hospital. Explanatory variables also included systemic infection, gender, APACHE II score, and age. For the test of misalignment, where no deaths occurred in the reference group, Firth's adjustment for complete separation in logistic regression as implemented by Heinze and Schemper was applied (30). All analyses were conducted using SAS Version 9.4 where a two-sided *p* < 0.05 was considered statistically significant.

## Results

### Feasibility of Detecting Diurnal Variation

**Figure 1:**
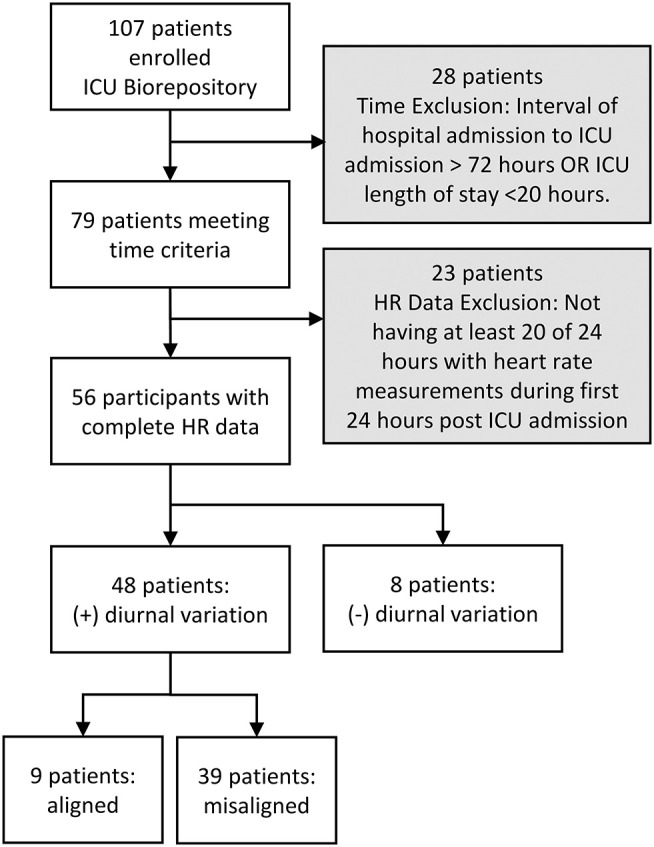
Consort diagram.

We screened 107 patients for inclusion in this study. Twenty-eight patients were excluded because they did not meet time criteria; an additional 23 (29%) out of 79 were excluded due to incomplete HR data. Thus, 56 patients were included in the initial cosinor test for diurnal variation of HR; 48 (86%) out of 56 had diurnal variation in HR (Figure 1).

### Patient Characteristics

Patients with diurnal variation in HR had an average (standard deviation) age of 63.3 years (13.3), an APACHE II of 17.8 (5.0), and had been in the hospital for a median of 7.5 h (IQR 4.7–14.2) prior to ICU admission (i.e., the start of recording). Notably, 10 (21%) of patients with diurnal HR variation were on vasopressors.

**Figure 2:**
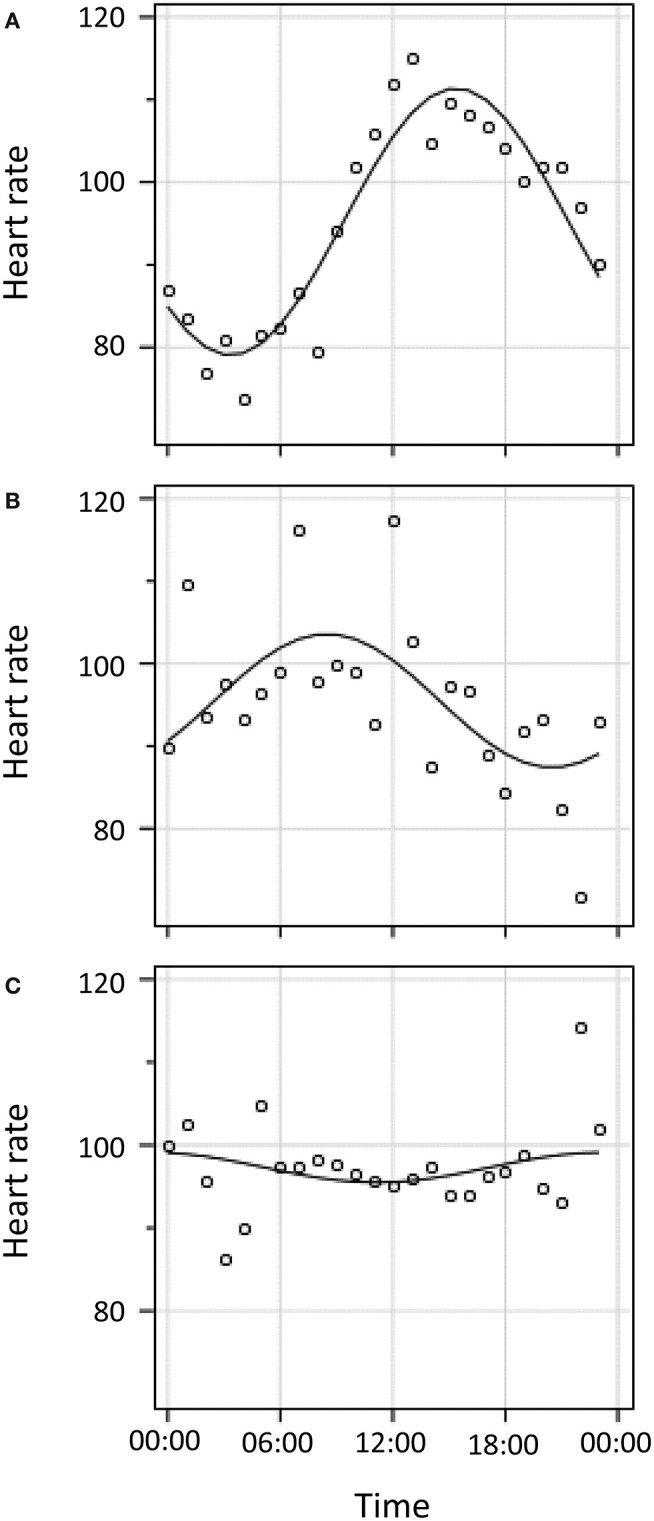
Cosinor curve fitting for three illustrative patients. **(A)**. Diurnal variation in HR with alignment of nadir to population norm of 04:00 (± 2 h). **(B)**. Diurnal variation in HR with mis-alignment to population norm of 04:00. **(C)**. No detectable diurnal variation in HR.

Table 1 presents additional patient characteristics presented in 3 groups which are illustrated in Figure 2: (1) patients with aligned diurnal variation, (2) patients with misaligned diurnal variation, and (3) patients without diurnal variation (not included in further HR analysis). BMI was statistically different among the 3 groups (*p* = 0.05). Other indicators of poor sleep and sleep disorders were not statistically different among groups. In fact, no other statistically significant differences between groups were observed.

**Table 1:** Demographic and critical illness patient characteristics.

**Characteristic**	**Patients with aligned diurnal variation (*n* = 9)**	**Patients with misaligned diurnal variation (*n* = 39)**	**Patients without diurnal variation HR (*n* = 8)**
Age: Years, Mean ± SD	63.0 ± 16.3	63.3 ± 12.7	56.1 ± 17.7
Non-white: *N* (%)	4 (44.4)	8 (20.5)	1 (12.5)
Women: *N* (%)	3 (33.3)	15 (38.5)	4 (50.0)
APACHE II: Mean ± SD	15.7 ± 3.8	18.3 ± 5.2	17.8 ± 6.1
Admitted from ER: N (%)	6 (66.7)	31 (79.5)	7 (87.5)
Time to ICU admission: Hours, Median (IQR)	8.3 (5.8–18.9)	6.5 (4.5–13.7)	10.4 (5.1–26.2)
Systemic infection: *N* (%)	3 (33.3)	11 (28.2)	3 (37.5)
Hospital LOS: Days, Median (IQR)	7.8 (5.8–10.3)	7.7 (4.7–14.5)	5.3 (4.1–12.0)
ICU LOS: Days, Median (IQR)	2.3 (2.0–2.6)	2.6 (1.8–4.1)	2.2 (1.3–2.8)
Death in hospital: *N* (%)	0 (0.0)	7 (17.9)	1 (12.5)
Delirium on admission: *N* (%)	2 (22.2)	7 (17.9)	3 (37.5)
BMI: Median (IQR)	27.5 (24.2–29.7)	28.8 (23.4–30.6)	33.7 (29.3–66.2)
PSQI “fairly bad” or “very bad” sleep: N* (%)	1/7* (14)	15/31* (48)	5/8* (63)
PSQI: frequent snoring: N* (%)	4/7* (57)	14/33* (42)	2/8* 25
Mechanical ventilation on admission: *N* (%)	1 (11.1)	2 (5.1)	1 (12.5)
Vasopressors on date of ICU admission: *N* (%)	1 (11.1)	9 (23.1)	25 (25.0)

*APACHE II, acute physiology and chronic health evaluation II; BMI, body mass index; ER, emergency room; HR, heart rate; ICU, intensive care unit; IQR, interquartile range; LOS, length of stay; PSQI, Pittsburgh Sleep Quality Index; SD, standard deviation*.

* *Not all participants were able to complete the PSQI; therefore, denominators are provided*.

All deaths occurred among those who either lacked diurnal variation or who were misaligned (p, non-significant).

### Diurnal Heart Rate Variation

The 48 patients with diurnal variation had a median mesor of 87 beats per minute (IQR 77–102), a median amplitude of six beats per minute (IQR 4–11), and a median nadir of 11.1 h past midnight (IQR 4.6–20.5 h). Of the 48 patients with diurnal HR variation, 39 were more than 2 h misaligned vs. the population norm of 04:00. Among patients who were misaligned, the mean misalignment of HR nadir was 6.1 ± 3.0 h. Of note, among patients with diurnal variation, all in-hospital deaths occurred among those with misalignment. There was also a trend toward an association between misalignment of diurnal variation in HR and increased severity of illness (non-significant *p* = 0.17).

### Association Between Misalignment and Death in Hospital

**Figure 3:**
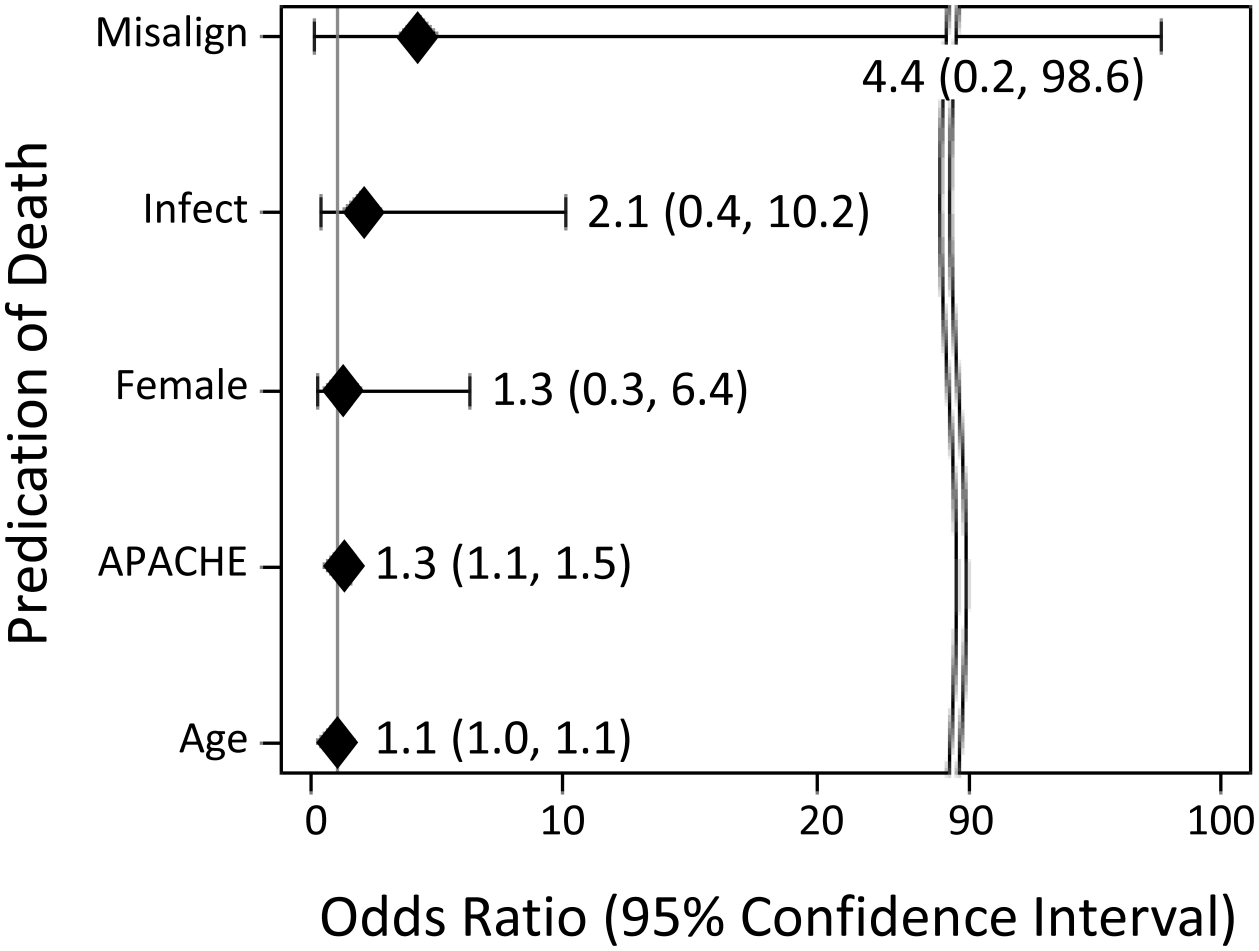
Unadjusted logistic regression testing the association between selected explanatory variables with death during the patient's stay in hospital. Explanatory variables include misalignment of diurnal variation (“misalign”), systemic infection (“infection”), gender (“female”), severity of illness as estimated by APACHE II (“APACHE”), and age in years (“age”).

In addition, in exploratory analyses, unadjusted logistic regression was used to evaluate the strength of association between selected explanatory variables with death during the patient's stay in hospital (Figure 3). The selected explanatory variables were as follows: misaligned diurnal variation of HR, systemic infection, gender, severity of illness, and age. The point estimate of misalignment's odds ratio (95% confidence interval) for death was 4.38 (0.2, 97.6). However, this association was not statistically significant.

## Discussion

It was feasible to detect diurnal variation in HR in a cohort of medical ICU patients. About three-quarters of patients had complete HR data. This number will continue to improve with ongoing refinements of our data capture system. Among critically ill patients with complete HR data, 86% had a detectable diurnal variation in HR. Notably, the vast majority of patients with diurnal variation were more than 2 h misaligned (before or after) relative to the population normal nadir of 04:00 (26). The ability to use diurnal variation in HR as a circadian proxy is important because current “gold standard” methods of circadian rhythm measurement (i.e., melatonin curves) are challenging and not practical in the ICU. In contrast, HR data is accessible and collected on most ICU patients as part of standard care.

Diurnal variation in HR is a well-established circadian phenomenon (27). In this cohort, diurnal HR variation was observed *despite* vasopressor use and other cofounders of HR. This finding is important since it could be hypothesized that the high frequency of vasopressor use in the ICU might lower the feasibility of using continuous HR as a circadian proxy.

Patients without diurnal variation in HR were noted to have a higher BMI; this raises the possibility that sleep-disordered breathing is associated with a lack of diurnal variation in HR. However, other sleep data such as subjective global sleep quality and report of habitual snoring did not support this conclusion. Because of the high prevalence of mechanical ventilation and supplemental oxygen use in this population, we could not use oxygen saturation or desaturation indices to estimate the presence of sleep-disordered breathing in our cohort.

There were no deaths among the patients with aligned diurnal HR variation. This difference was not statistically significant which we attribute to both small sample size and the analytic limitations that were caused by all deaths occurring in the misaligned diurnal variation patient group. However, this exploratory finding could reflect an important biologic signal. It is plausible that circadian misalignment contributes to poor recovery and contributes independently to death. Other studies have observed that disruptions of the sleep and circadian systems are associated with higher mortality in medical ICU patients (31–33) and other critical illness populations (34–37). Conversely, we note that is a simple association rather than any implied causal effect, and this misalignment may be a marker of severity of illness. We also observed a trend toward higher severity of illness in the misaligned group in our data.

### Limitations

As a feasibility pilot, the primary limitation of this study was its small sample size, which strongly limited its ability to test for associations with medical ICU outcomes. However, given the demonstrated feasibility, this type of data collection can now be leveraged in larger studies moving forward. In addition, because most patients in our cohort were transferred out of the medical ICU shortly after the 24-h timeline, we had very few patients with 48 or 72 h of data. Therefore, we were unable to analyze changes in diurnal variation after the first 24 h of medical ICU admission. Finally, this data and diurnal variation was not compared to markers of the central circadian clock (e.g., melatonin).

### Future Directions

Future studies will directly address the above listed limitations. A larger prospective study inclusive of central circadian measurements would allow further validation of this circadian marker and test more definitively for associations between death and circadian misalignment. Furthermore, a large study would include more patients admitted to the medical ICU for longer than 24 h which would allow the analysis of changes in diurnal variation over longer periods in the medical ICU.

## Conclusions

We have shown that it is feasible to assess diurnal variation of HR in medical ICU patients. There are important trends in association with death and severity of illness. Future work will validate this a marker of circadian rhythmicity and test further for associations with medical ICU mortality.

## Data Availability Statement

The datasets generated for this study are available on request to the corresponding author.

## Ethics Statement

The studies involving human participants were reviewed and approved by Yale University Institutional Review Board. The patients/participants provided their written informed consent to participate in this study.

## Author Contributions

MK designed and conducted the study; she assisted in all statistical analyses and wrote the manuscript. MD and TM conducted all statistical analyses and edited the manuscript. NR, MP, and HY guided design, conduct, and analysis of the study and edited the manuscript. All authors contributed to the article and approved the submitted version.

## Conflict of Interest

The authors declare that the research was conducted in the absence of any commercial or financial relationships that could be construed as a potential conflict of interest.
